# CIGene: a literature-based online resource for cancer initiation genes

**DOI:** 10.1186/s12864-018-4944-y

**Published:** 2018-07-25

**Authors:** Yining Liu, Mingyu Luo, Qijun Li, Jiachun Lu, Min Zhao, Hong Qu

**Affiliations:** 1 School of Public Health, Institute for Chemical Carcinogenesis, Guangzhou Medical University, 195 Dongfengxi Road, Guangzhou, 510182 China; 2 School of Engineering, Faculty of Science, Health, Education and Engineering, University of the Sunshine Coast, Maroochydore DC, Queensland 4558 Australia; 3 Center for Bioinformatics, State Key Laboratory of Protein and Plant Gene Research, College of Life Sciences, Peking University, Beijing, 100871 People’s Republic of China; 4 School of Public Health, The First affiliated Hospital, Guangzhou Medical University, Guangzhou, 510120 China

**Keywords:** Cancer initiation gene, Network analysis, Cancer genomics, Functional analysis

## Abstract

**Background:**

Cancer initiation genes (CIGs) are genes that can directly promote cell proliferation or induce cancer. There are thousands of published studies identifying various CIGs; however, no systematic collection or description is available.

**Results:**

To construct a CIG reference for genetic screening, we have collected 177 human genes curated from 1507 PubMed abstracts. To facilitate data queries and browsing, the identified CIGs along with extensive bioinformatic annotations were stored in an online database called CIGene. Initial functional analysis revealed an overlooked role for cell motility in cancer initiation. Subsequent cross-referencing of known tumor suppressor genes and oncogenes against the 177 CIGs identified 96 and 81 CIGs with and without known oncogenic roles, respectively. Successive network analyses of all 177 CIGs determined that the two groups of genes were more likely to link within their group. The distinct molecular functions for these groups were also confirmed with functional studies. While the 96 known oncogenic genes had fundamental roles in gene regulation and signaling, the remaining 81 genes possessed more ancillary functions, such enhancer binding. Further network and mutational analysis of the 96 known oncogenic genes revealed that mutations in these genes were highly prevalent in multiple cancers. By focusing on breast cancer, we found that 32 of the 96 genes with mutations in breast cancers were significantly associated with patient survival.

**Conclusions:**

As the first literature-based online resource for CIGs, CIGene will serve as a useful gateway for the systematic analysis of cancer initiation. CIGene is freely available to all academic users at http://soft.bioinfo-minzhao.org/cigene/.

**Electronic supplementary material:**

The online version of this article (10.1186/s12864-018-4944-y) contains supplementary material, which is available to authorized users.

## Background

Cancer development is a dynamic cellular process driven by multi-step acquisition of genetic mutations, clonal expansion, and selection [1]. The most critical step is the establishment of initiating cells (clones), which lead to sub-clonal diversification and adaption resulting in uncontrolled growth [2]. Though numerous mutations may occur during cancer progression, resulting in genetic heterogeneity, the somatic mutations that initiate cancer inadvertently provide potent selective pressure for the expansion of cancer clones [1]. Therefore the identification of cancer initiation genes (CIGs) within and across tumor types is critical for cancer diagnosis and for the development of personalized therapies.

Genetically, the stepwise acquisition of defective tumor suppressor genes (TSGs) and hyperactive oncogenes (OCGs) greatly contribute to the dysregulation of cell proliferation and apoptosis during oncogenesis [3]. Since TSGs and OCGs influence cancer cell growth in opposite ways, they often exist as competitive controls on cancer progression [4]. To date, thousands of potential cancer-related genes, including TSGs and OCGs, have been identified in various cancer types; however, mutations in these genes could be acquired during cancer progression and might not have a specific role during cancer initiation.

Over the past years, a number of genetic and epigenetic approaches have been used to identify CIGs for various cancers [5]. Thus, we set out to generate a collection of CIGs from the literature to further our understanding of their function and to evaluate their roles among different cancers. Our aim was to organize a collection of CIGs and build the first literature-based CIG database, CIGene. With the ability to cross-reference with large-scale cancer expression and prognosis databases, CIGene will serve as a valuable resource to efficiently define cancer initiation events, including somatic mutations, gene regulation, and gene interactions. In total, we isolated 96 CIGs with known tumor suppressive or oncogenic roles, and these genes may define an important network involved in cancer initiation. Additionally, we identified 81 novel CIGs without any known tumor suppressor or oncogene roles, providing new candidates for developing novel cancer diagnostic strategies.

## Methods

### Literature curation

We collected CIGs from the GeneRIF and PubMed literature databases by searching the following expression: “initiation [title] or initiating [title] or initiator [title]” and “cancer or tumor or carcinoma or adenocarcinoma or craniopharyngioma or glioblastoma or insulinoma or leukemia or lipoma or lymphoma or melanoma or myoma or neoplasm or nephroblastoma or retinoblastoma or sarcoma”. In all, 1507 relevant entries were retrieved from PubMed (as of November 4, 2016), including the PubMed ID, gene name, and the sentences with matched keywords. We then manually checked all of the sentences and the corresponding PubMed abstracts to identify the correct CIGs within the text. All Entrez gene IDs were identified by searching for the gene name mentioned in the literature. For nonhuman genes, we mapped them to their corresponding human homologues using the NCBI HomoloGene database [6].

### Database construction

To further annotate the identified genes, we integrated additional annotations from the Entrez Gene database, including Gene ID, official symbol, gene aliases, chromosome location, functional description, gene ontology (GO), and related pathways. The general information and homologous sequences are crosslinked to the NCBI Entrez and HomoloGene databases [7]. The mRNA expression profile data from both normal and tumor tissues were imported from BioGPS [8]. To obtain comprehensive pathway-related information, we annotated the genes using BioCyc [9] and the KEGG collection of databases [10]. The other useful regulatory information included post-translational modification [11], methylation [12], and protein-protein interactions [13]. All of the included functional and/or genomic features were seamlessly integrated to produce a downloadable output available in a plain text format.

All of the literature and annotation data are stored in a MySQL-based database for CIGene. CIGene offers two data search functions: text-based query and sequence-based BLAST search. The text-based query retrieves a list of CIGs with annotations of interest. The sequence-based BLAST is for annotating genes with DNA or amino acids sequences. Furthermore, a browsing function was implemented in a variety of ways, including access to annotated gene information from the TSGene database [14], highlighted KEGG pathway maps, and genomic positions.

### Functional enrichment analysis

Gene set enrichment analysis was conducted by submitting a list of genes of interest to the ToppFun online server [15]. ToppFun is a web server that allows users to explore gene annotations, such as chromosome locations, associated diseases, protein domains, molecular functions, cellular components, biological processes, and pathways. The database is updated regularly and annotates all input genes with biological pathway, GOs, protein family, and other predetermined functional annotations from various resources. To calculate statistical *P*-values for those enriched annotations for the input genes, a collection of all human protein-coding genes was used as the baseline. Subsequently, a hypergeometric test was applied to each annotation term following multiple test corrections using the Benjamini-Hochberg method. The corrected P-values were used to filter out irrelevant annotations using the threshold value of *P* < 0.01. Furthermore, over-representative GO terms were visualized using the REVIGO online server [16].

### Network and mutational analyses

To further explore the connection of CIGs to other cancer genes, we separately mapped 96 and 81 CIGs with and without oncogenic and tumor suppressive roles, respectively. To this end, we downloaded a non-redundant human interactome from the PathCommons database [13], containing 3629 proteins and 36,034 protein-protein interactions. It is of note that we only used those protein-protein interactions collected from pathway databases (HumanCyc, Reactome, and KEGG pathway) [10, 17], which have clear biological significance, rather than physical interactions without experimental validations. By using a sub-network extraction pipeline implemented in our previous study [18], we build a sub-network to link the CIGs with other human genes based on those pathway-based interactions. Briefly, the CIGs were mapped into the prepared pathway-based interactome and the sub-network was extracted according to the shortest paths between those input CIGs and other genes. By calculating the topological properties of the sub-network using the Network Analyzer plugin in Cytoscape 3.4 [19], we were able to explore the potential global network properties of cancer initiation [20]. Here, the node degree distribution was used to characterize the total number of connections for each gene in the network [20], and the shortest path was calculated using the shortest length from one node to another [20]. All network visualization was drawn using Cytoscape 3.4. The node degree was used to depict the node size in the network chart. Also, different colors were used to differentiate those CIGs from our input and the other linker genes bridging those CIGs. The mutational analyses were all conducted using the cancer genomics portal cBio [21].

## Results

### Data collection and web interface for the CIGs database

To survey all known CIGs, we performed extensive literature curation utilizing the GeneRIf and PubMed databases. We then extracted the relevant sentences by keyword matching. By carefully curating all the sentences, we identified all CIGs clearly defined within the literature. For nonhuman studies, we mapped the identified genes to their human homologs using the NCBI HomoloGene database. By formalizing the gene alias to the official gene symbol we could then map the curated genes to a public human gene database and integrate functional annotations. The final stage of our literature curation resulted a list of 177 non-redundant CIGs Additional file 1: (Table S1).

**Fig. 1:**
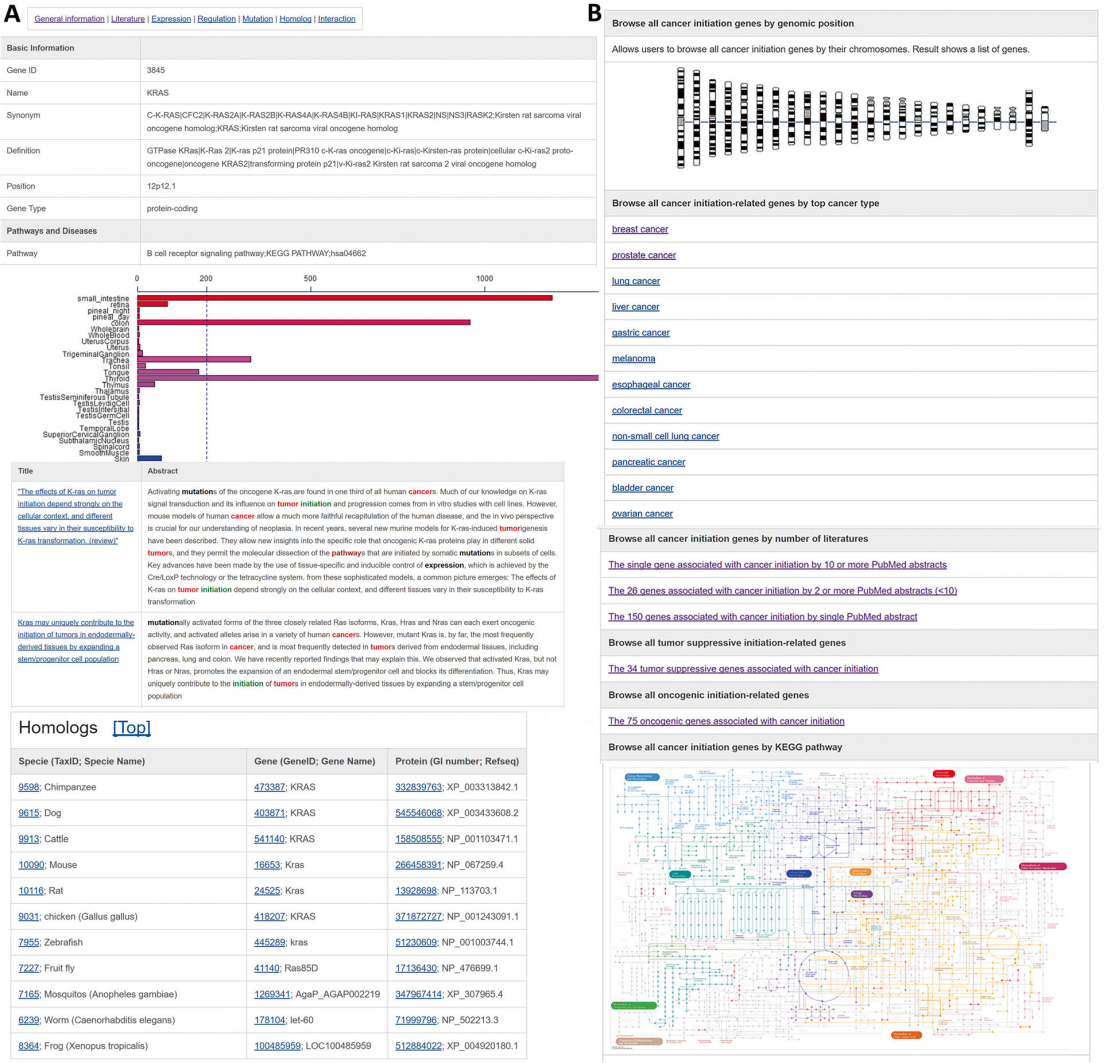
CIGene web interface. **a** The basic information on each CIG page. **b** Browsing the genes in CIGene using chromosomes, cancer sub-types, literature support, tumor suppressor, oncogenes, and KEGG pathways

After importing all the curated CIGs and annotations into the database, we then developed a user-friendly web interface to query all the information. As shown in Fig. 1, a typical gene entry in the CIGene database contains seven categories of information, assessed by clicking the labels at the top, including General information, Literature, Expression, Regulation, Mutation, Homolog, and Interaction. Marked abstracts of the curated papers are provided in the “Literature” page. The “Homolog” page is used to map the human CIGs to other model species, including mouse, rat, zebrafish, and fruit fly. Additionally, the “Regulation” page provides regulatory information about the genes, including interactions with transcription factors, post-translational modification, and methylation. Importantly, all of the information, including functional features, is formatted in plain text for downloading. Together, these comprehensive annotations and the pre-computed regulatory information provide a basis for the systematic analysis of CIGs to aid in the elucidation of cancer initiation.

### Biological pathways associated with cancer initiation

To define the molecular functions and mechanisms of CIGs, we utilized functional enrichment analysis to identify over-represented biological information within the list of genes of interest based on *P*-values. The initial analysis included the annotation of GO terms and KEGG pathways. Subsequently, hundreds of hypergeometric tests within the set of genes were conducted, generating hundreds of *P*-values. Using Benjamini-Hochberg correction, we further adjusted the enrichment analyses and identified those pathways and disease entries with adjusted *P*-value less than 0.01 Additional file 2: (Table S2). In total, we identified 2470 pathway or GO terms significantly associated with the 177 CIGs. This identified the critical roles of CIGs as being involved in a majority of basic cellular processes. Some enriched pathways and functional terms were cancer related and included “proteoglycans in cancer” (adjusted P-value = 3.96E-22), “pathways in cancer” (adjusted P-value = 1.46E-30), “microRNAs in cancer” (adjusted P-value = 1.30E-16), and “ras pathway” (adjusted P-value = 7.45E-16). Notably, 47 CIGs were shown to be associated with the positive regulation of locomotion, cell motility, cellular component movement, and/or cell migration (all adjusted *P*-values < 0.01; Additional file 2: Table S2).

### Identification of a novel cancer initiation mechanism by mapping to known tumor suppressors and oncogenes

**Fig. 2:**
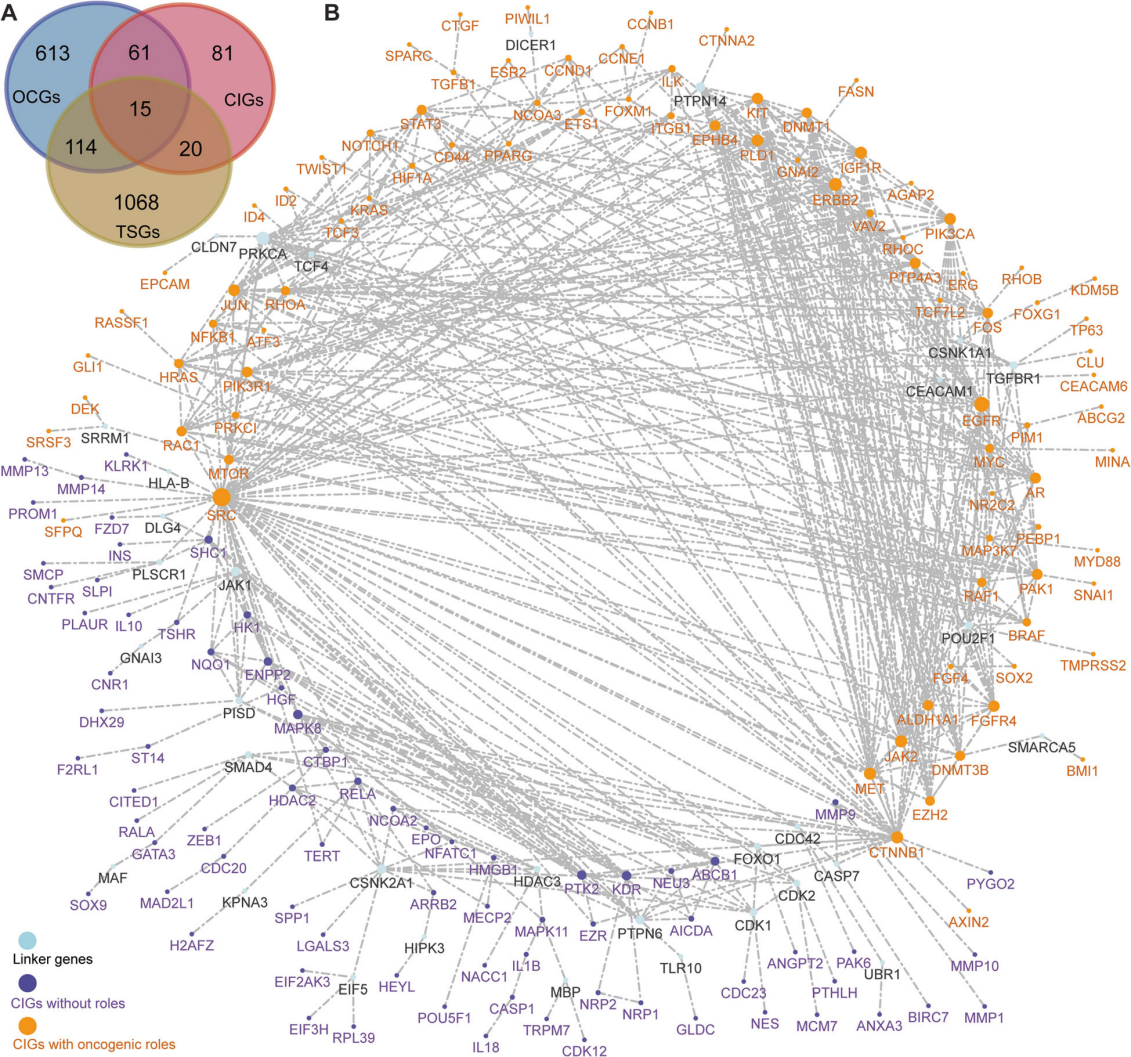
CIGs with tumor suppressor and oncogenic roles. **a** Venn diagram of three cancer-associated gene datasets: cancer initiation genes, oncogenes, and tumor suppressors. **b** A reconstructed network with 177 CIGs

To further explore the roles of CIGs in cancer progression, we cross-referenced the genes in our curated database with known OCGs [22] and TSGs [3]. This analysis identified 96 CIGs reported as either OCGs or TSGs (Fig. 2a). To assess the associated cellular events of these CIGs at the system level, we constructed the first interactome related to CIGs (Fig. 2b). Since many CIGs are related to fundamental cellular processes, they may possess thousands of predicted protein interaction partners. To focus on those interactions of biological significance, we only include the most reliable interactions from curated resources, such as the KEGG and Reactome databases, as previously implemented [3]. A subnetwork of CIGs was then extracted from all of the human-based interactomes. The reconstructed CIG interactome contained 196 genes and 567 gene–gene interactions based on current evidence from known biological pathways (Fig. 2b). Of the 196 nodes, 161 were CIGs and the remaining 35 were genes that potentially bridge CIGs to fully implement a given cellular function. By categorizing the TSGs and OCGs, we found the 96 CIGs with known tumor suppressor or oncogenic roles are more likely to connected compared to the remaining 81 CIGs without any defined roles as TSGs or OCGs. This observation suggests that these two groups of CIGs may have distinct functions in cancer initiation (Fig. 2b).

**Fig. 3:**
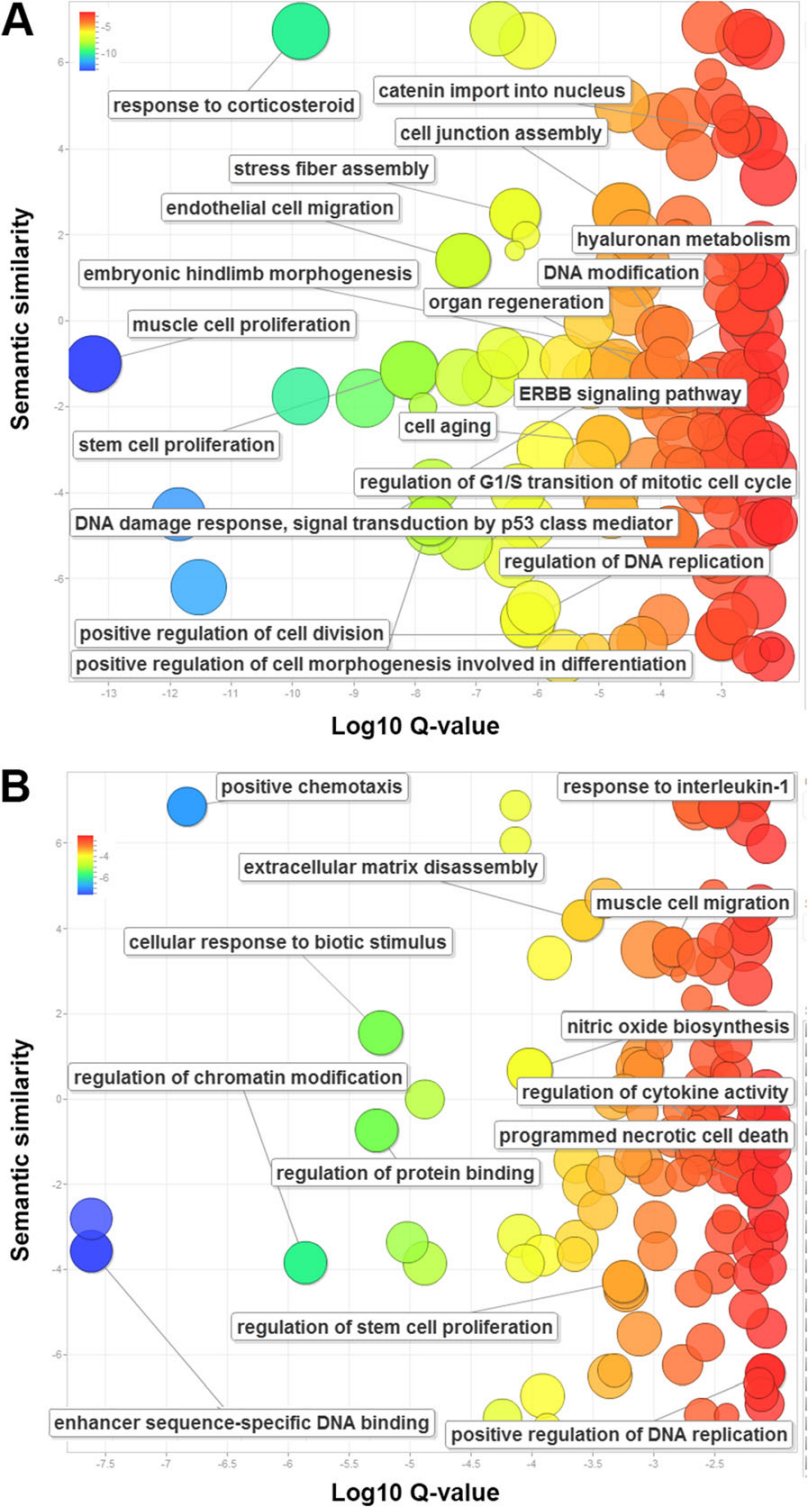
The enriched gene ontology (GO) terms for the 96 genes with tumor suppressor and oncogenic roles (**a**) and the remaining 81 genes (**b**). The scatterplot presents the summarized GO terms. Circles indicate the GO clusters and are plotted in two-dimensional space according to other GO terms’ sematic similarities. X-axis shows the log10 *P*-value; the small corrected *p*-values are plotted in the left. The circle colour represents the same information of log 10 P-value; the blue direction implies the smaller *P*-values. Y-axis represents semantic similarities of gene ontology terms

To further explore the differences between the two CIG groups, we conducted the functional enrichment analyses separately (Fig. 3a-b). For the 96 genes that intersected with TSGs or OCGs, we found 2380 over-represented pathways or GO terms based on adjusted *P*-values < 0.01 Additional file 3: (Table S3), and among these functional terms, 715 and 350 contain the keyword “regulation” or “signaling,” respectively. The top over-represented signaling transduction terms included enzyme linked receptor protein signaling pathway, transmembrane receptor protein tyrosine kinase signaling pathway, Rap1 signaling pathway, signaling by interleukins, ErbB1 downstream signaling, signaling events mediated by hepatocyte growth factor receptor (c-Met), signaling pathways regulating pluripotency of stem cells, etc. In addition, these genes were involved in many fundamental processes, including stem cell proliferation, cell aging, regulation of cell cycle and division, cell junction, and migration (Fig. 3a). We additionally found a number of novel pathways, such as response to corticosteroid (adjust *P*-value = 6.34E-13), with corticosteroids representing a class of steroid hormones with anti-inflammatory, immunosuppressive, anti-proliferative, and vasoconstrictive effects.

Additionally, the 81 genes that did not map to known OCGs or TSGs may provide novel insights due to their relevance to cancer initiation. Enrichment analysis associated the 81 genes with 482 pathways and GO terms (Additional file 4: Table S4; all adjusted *P*-values < 0.01). Many of these 81 genes associated with over-represented cancer-related processes, such as positive regulation of angiogenesis and regulation of stem cell proliferation (Fig. 3b). Furthermore, these genes also represented key regulators of gene expression, including regulation of chromatin modification (adjust P-value = 1.39E-06) and enhancer sequence-specific DNA binding (adjust P-value = 7.38E-05). In addition, the regulation of cytokine secretion (adjust P-value = 1.33E-05) and response to interleukin-1 (adjust P-value = 3.44E-03) were also associated with these genes, indicating novel aspects of extracellular regulation may be involved in cancer initiation.

### Network and mutational analysis of 96 CIGs with known tumor suppressor or oncogenic roles reveal important hub genes associated with patient survival

**Fig. 4:**
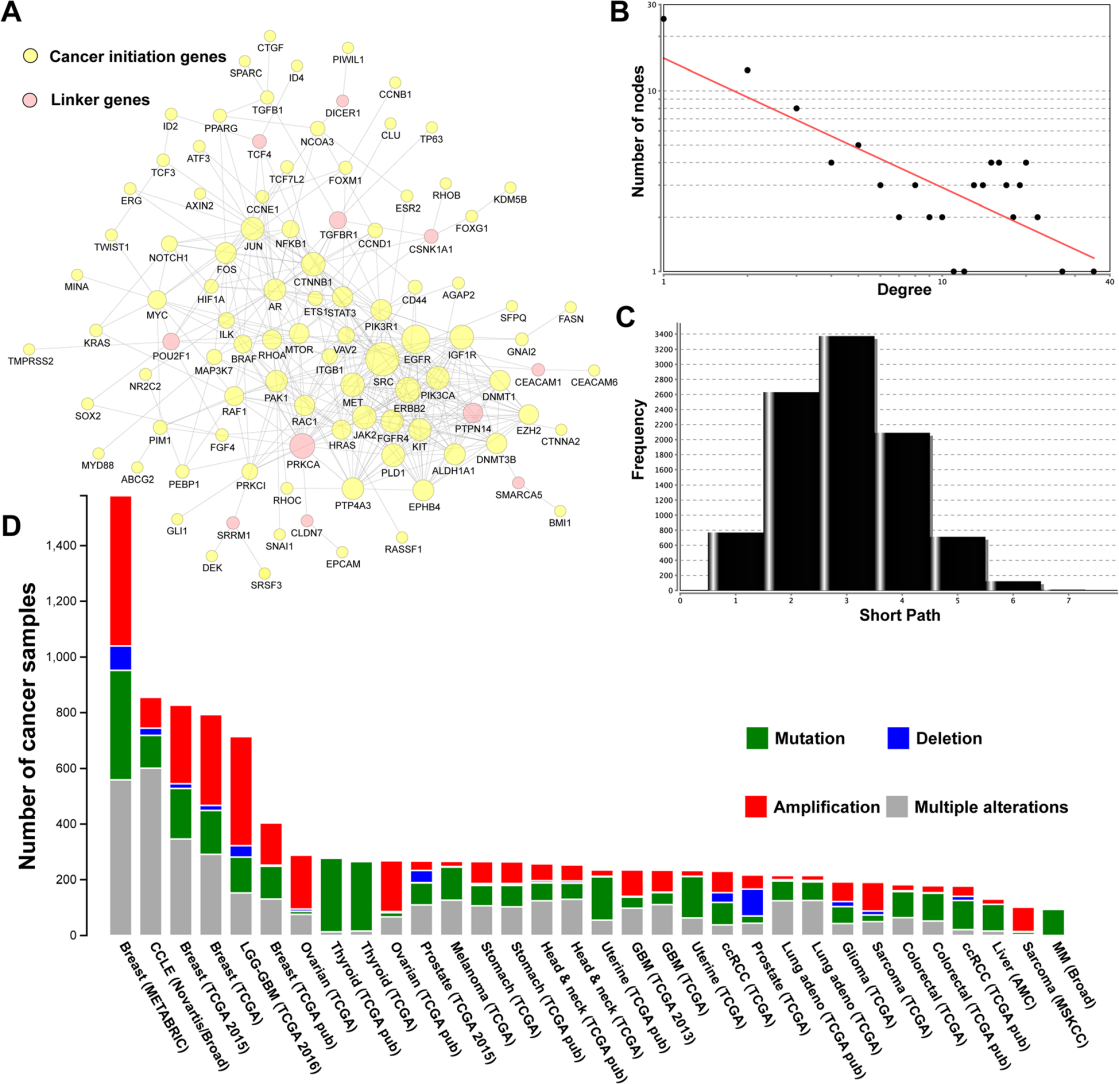
The CIGs interactome generated from pathway-based protein-protein interaction data. **a** The 88 genes in yellow are the CIGs; the remaining 11 genes in pink are linker genes that connect the 88 CIGs. The size of the nodes represents the number of connections in the network, **b** the degree distribution, and (**c**) the path length frequency. **d** Mutation map of the 96 CIGs in all cancers. X axis: cancer types in TCGA. Y axis: altered counts of the 96 genes that correspond to the cancers represented on the X axis. The different colors indicate different cancer types based on their tissues of origin

From our analysis of the 96 CIGs that mapped to known OCGs or TSGs, we extracted a sub-network containing 99 genes and 382 gene–gene interactions (Fig. 4a). Of the 99 nodes, 88 were CIGs; the remaining 11 were linker genes that may potentially bridge CIGs to fully implement their cellular functions. The majority of the CIGs were linked to each other in a highly modular structure, and this was concluded by comparing the exponent value with that of whole human PPI network. The connections of all nodes in the biological network often follow a power law distribution *P*(*k*)*~k*^*-b*^, where *P*(*k*) is the probability that a gene has connections with other *k* genes and *b* is an exponent with an estimated value. For the sub-network of CIGs, the degree (the number of connections) and number of nodes followed an exponential function of y = 15.14*x*^−0.716^ (*R*^2^ = 0.697; Fig. 4b), while most nodes in human PPI networks are sparsely connected with the exponent value of 2.9 [23]. Moreover, the characteristic path length of the CIG sub-network was 3, indicating only three steps between most node connections, which is substantially smaller than the average for the human PPI network (Fig. 4c). This finding supports the accuracy of our data and also shows that the 96 CIGs are highly interconnected and form a high-density cellular modulus. By prioritizing the node degree for all of the genes, we found a number of genes with high connectivity acting as hub genes, including *SRC*, *PIK3CA*, *ERBB2*, and *EGFR*.

Using published TCGA mutational data, we produced a mutation map across various cancers for the 96 interconnected CIGs. This analysis was conducted using cBioPortal (Fig. 4d). To ensure reliability, the cancer types with a total number of samples of less than 50 were excluded. The CIGs demonstrated high variation counts across multiple cancers, including breast cancer, brain cancer, ovarian cancer, thyroid cancer, and prostate cancer. These results confirmed the importance of the initial genes in cancer development, implying their potential application for clinic diagnosis.

**Fig. 5:**
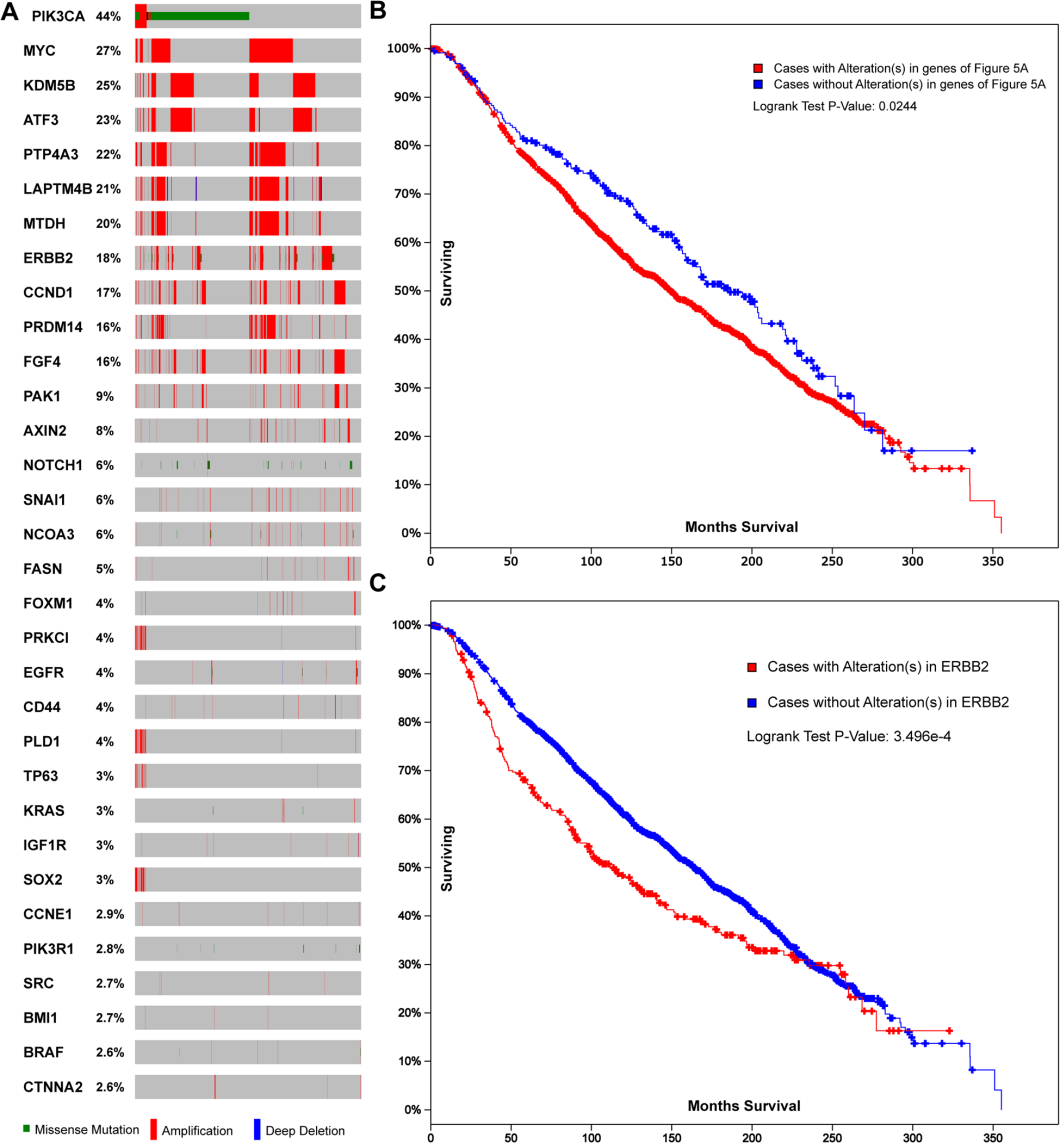
Sample-based mutations of the 96 CIGs in the METABRIC breast cancer dataset. **a** OncoPrint for breast cancer. Only genes with a mutation rate over 0.5% are shown in the figure. Multiple alterations: more than one type of mutation. Survival analyses for all 32 genes (**b**) and ERBB2 (**c**). Survival characteristics of the genes in (**a**)

We chose to further focus on breast cancer, as it had the most mutational correlation with the 96 CIGs. We plotted the sample-based mutational profile to find the genes within this group with a high mutation rate (Fig. 5a). The breast cancer dataset included 1904 tumor samples/patients with sequencing and copy number variance (CNV) data; genetic alterations were discovered in 83% of cases (1580 out of 1904). In total, we found 32 genes to have genetic alterations in the breast cancer cohort. Some top mutated genes included *PIK3CA*, *MYC*, *KDM5B*, *ATF3*, *PTP4A3*, *LAPTM4B,* and *ERBB2*, with alternation rates of 44, 27, 25, 23, 22, 21, 20, and 18%, respectively (Fig. 5a). Subsequent survival analysis revealed statistically different survival times between the patients with or without any mutation in these 32 genes (Fig. 5b).

Interestingly, though *PIK3CA* had a very high mutational rate among all cohorts, the survival analysis indicated no substantial difference between the patients with or without *PIK3CA* mutations. This may indicate that though the *PIK3CA* gene mutation is important to the occurrence of breast cancer it is not influential to the survival time of patients. In contrast, the patients with the *ERBB2* mutation had substantially different surviving curves, with a sharper decreasing survival rate at the early period and a shorter median survival in months compared to patients without this genetic alteration (Fig. 5c). Taken together, these results highlight the potential roles of these CIGs in the progression of breast cancer.

We also built a network for the 81 novel CIGs without oncogenes and tumor suppressive roles Additional file 5: (Table S5). The network may reveal a hierarchical regulatory mechanism between those CIGs and some linker genes. Among the input 81 genes, 77 CIGs are connected by 22 linker genes. Interestingly, some of genes are in the top regulatory level with multiple connections such as *EZR*, *AICDA*. Both genes are frequently detected in the early-stage cancer, which may imply their important roles for cancer initiation.

To further evaluate the significance of our curated 177 CIGs related to the cancer survival, we applied an empirical re-sampling approach on the precomputed survival correlation results in 21 TCGA cancer types. Here, we take the TCGA Lower Grade Glioma (LGG) dataset as an example. First, we counted how many CIGs are significantly correlate with LGG patient survival based on the precomputed Cox analysis (*P*-values < 0.05) from oncoLnc database [24]. The number of these survival-related genes for our 177 CIGs is 102. Next, for all the 18,616 genes with survival analysis results from oncoLnc, we randomly selected 177 genes and checked over the number of genes with significant correlation with patient survival (P-values < 0.05). The randomization processes were repeated for 10,000 times. Then, we counted the number of random selected node sets (N) whose number of survival-related genes was higher than the observed 102 in our 177 CIGs. Finally, an empirical P-value was calculated based on the N/10000 for the LGG dataset. The similar approaches were applied to all the remaining 20 TCGA cancer types and the final summarized *P*-values on all the 21 cancer types were provided in Additional file 6: Figure S1. As shown in the Table, all the P-values are less than 0.05 except the Pancreas adenocarcinoma (PAAD) dataset (P-value = 0.091). This result supports that our collected 177 CIGs are significantly related to cancer survival comparing to any random selected genes in TCGA pan-cancer data.

## Discussion

In this study, we constructed the first literature-based CIGene database, which currently contains 177 human genes curated from 1507 PubMed abstracts. CIGene has user-friendly interface, which can provide users with inquiries about functional and genomic features of CIGs.

Our systematic analysis associated CIGs with cell motility. Intuitively, cell motility is important for cancer invasion and metastasis [25]; however, by reviewing the literature to date, we found that the critical role of cell motility in cancer initiation has been overlooked [26]. Moreover, the prolactin-signaling pathway was also identified as another overlooked aspect of cancer initiation, which was highly enriched in our CIGs (adjusted P-value = 2.07E-20). Although prolactin has a well-known role in lactation, recent studies have reveal that the 16 kDa isoform derived from native prolactin has inhibitory effects on angiogenesis and tumorigenesis in breast and prostate cancers [27]. Thus, our systematic literature curation was able to identify diverse biological roles for CIGs, including a number of novel biological processes not previously explored in cancer initiation.

Furthermore, by overlapping 177 curated CIGs with the OCGs and TSGs datasets, we found that 96 CIGs with fundamental roles in cancer initiation associated with oncogenic or tumour suppressive processes. Additional mutation analysis revealed that mutations in 96 CIGs are highly prevalent in multiple cancers. It was also found that among the 96 CIGs, 32 genes with higher mutation rates were significantly associated with patient survival. Further functional enrichment analysis for the 96 CIGs and the remaining 81 CIGs may represent novel mechanisms involved in the process of cancer initiation such as non-coding RNA related mechanisms [28].

## Conclusions

In conclusion, our systematic curation of the literature related to cancer initiation yielded 177 CIGs in humans. By cross-referencing known cancer genes, we explored the functional, network, and mutational features of those CIGs with and without tumor suppressor or oncogenic roles. Since most these CIGs themselves were highly interconnected, only a few linking genes were added to construct a complete network. As such, special attention in future analyses should be paid to the linking genes *PRKCA*, *PTPN14*, *POU2F1*, and *TGFBR1*, because of their possible roles in cancer initiation. The complete list of CIGs available in the CIGene database provides a resource for cancer researchers to perform high-throughput genetic and clinical screens to aid in cancer diagnosis and treatment.

## Additional files

Additional file 1: **Table S1.** Details of the 177 human CIGs. (XLS 46 kb)

Additional file 2: **Table S2.** Functional enrichment analysis for all 177 CIGs. (XLS 393 kb)

Additional file 3: **Table S3.** Functional enrichment analysis for the 96 CIGs with identified tumor suppressor or oncogenic roles. (XLS 377 kb)

Additional file 4: **Table S4.** Functional enrichment analysis for the 81 CIGs without identified tumor suppressor or oncogenic roles. (XLS 100 kb)

Additional file 5: **Table S5.** Empirical *P*-values of survival significance of CIGs in 21 TCGA cancer types. (XLS 33 kb)

Additional file 6: **Figure S1.** The interactome for the 81 novel CIGs without oncogenic and tumor suppressive roles. The 77 genes in purple are the CIGs; the remaining 22 genes in green are linker genes that connect the 77 CIGs. (TIF 3130 kb)

## Abbreviations

CIG: Cancer initiation gene
CNV: Copy number variance
TCGA: The Cancer Genome Atlas
