# Supplementary figures and images for: CIGene: a literature-based online resource for cancer initiation genes
Source: BMC Genomics. 2018 Jul 25;19:552. doi: 10.1186/s12864-018-4944-y (PMC6060465; doi:10.1186/s12864-018-4944-y)

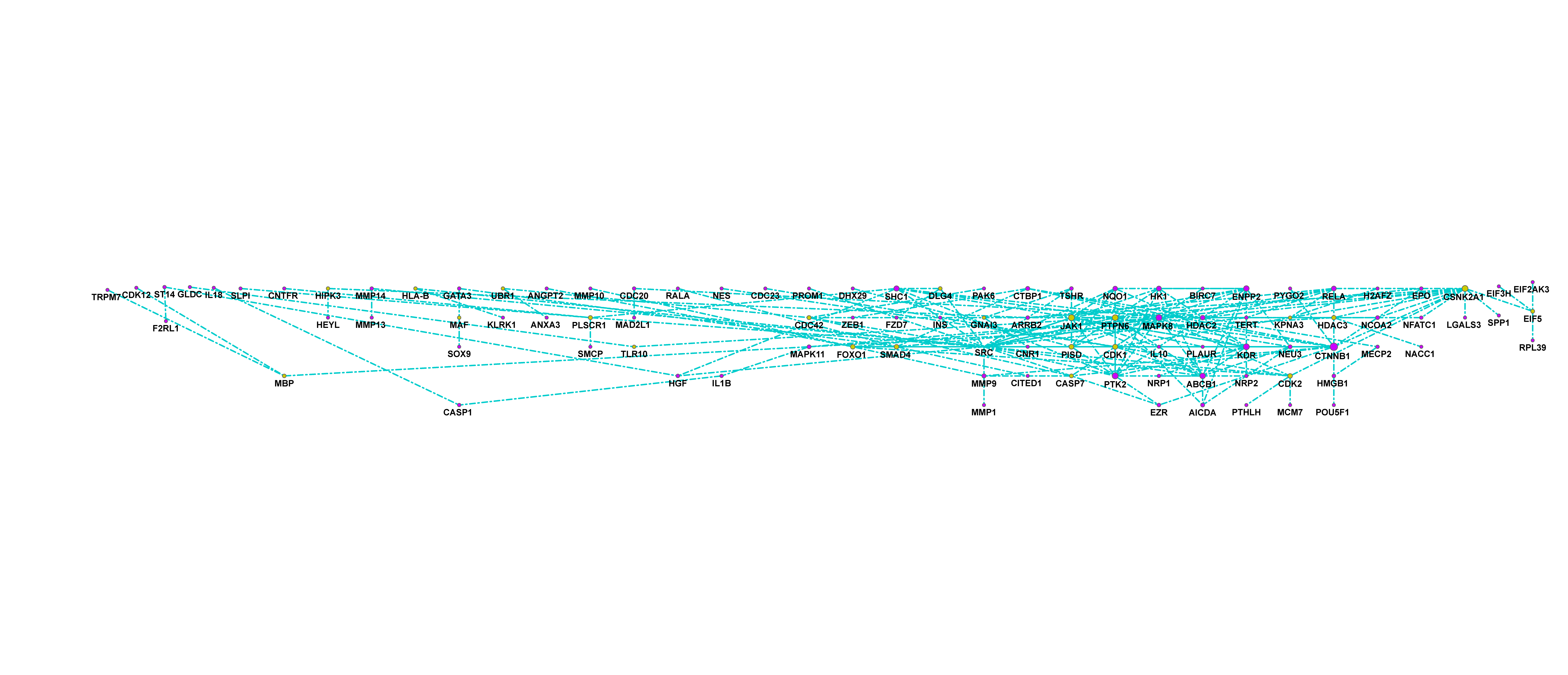

Supplement: Supplementary file 6 — Figure S1. The interactome for the 81 novel CIGs without oncogenic and tumor suppressive roles. The 77 genes in purple are the CIGs; the remaining 22 genes in green are linker genes that connect the 77 CIGs. (TIF 3130 kb) [file 12864_2018_4944_MOESM6_ESM.tif]
